# CAR T-Cell Therapy in Cancer: Balancing Efficacy with Cardiac Toxicity Concerns

**Published:** 2025-09-08

**Authors:** Parth Bhargava, Devendra K. Agrawal

**Affiliations:** Department of Translational Research, College of Osteopathic Medicine of the Pacific, Western University of Health Sciences, Pomona, California 91766 USA

**Keywords:** B-cell acute lymphoblastic leukemia, Cancer, Cardiotoxicity, CAR-T cell therapy, Cytokine release storm, Diffuse large B-cell lymphoma, Gastro-esophageal cancer, Mantle cell carcinoma, Multiple myeloma, Non-Hodgkin’s B cell lymphoma, Pancreatic cancer, Primary mediastinal large B-cell lymphoma

## Abstract

CAR-T therapy (Chimeric antigen receptor T-cell therapy) is a novel and transformative approach to fighting cancer, modulating the immune system involving adoptive cell therapy. In this article, a comprehensive information is provided on the effectiveness of CAR-T therapy in various types of cancers some of which have shown to be particularly challenging cancers to fully eliminate. The clinical effectiveness of CAR-T therapy compared to other traditional methods of cancer treatments such as surgical, radiotherapy, high dose rate (HDR)-brachytherapy, and immunotherapy favors CAR-T cell treatment strategy. The article presents the concept of CAR-T, discuss the structure and function of this novel therapeutic and its efficacy in liquid cancers, and development of therapies in solid cancers. Partial and complete response rates in relation to the CAR-T methodologies on various cancers are critically reviewed with discussion on the durability and overall long-term effects and limitations. Potential role of CAR-T therapies in various cancer treatments is reviewed in conjunction with other therapies and the efficacies and status on the progress of those therapies. Despite the impressive efficacy of CAR-T therapy, it is associated with potentially serious side effects, particularly cardiovascular toxicities that require proper cardiovascular screening prior, during, and after CAR-T therapy. These highlight the standardized modes of monitoring, prevention, and management of the toxicities. The cytokine release storm (CRS) in the pathogenesis of CAR-T toxicities may manifest in different ways emphasizing heterogeneous clinical presentation of CAR-T adverse events, highlighting the need for vigilant monitoring and individualized management strategies. Nonetheless, additional information on the serious adverse effects and long-term effects warrants further investigation.

## Introduction

Cancer remains as one of the leading causes of death globally where roughly one in six deaths may occur due to the disease. Cancer remains as a top three causes of death in patients aged 30–69 years in 177 of 183 countries [1–4]. This effect was exacerbated due to the COVID-19 pandemic which slowed down cancer diagnoses by 2 to 3 years [5–10]. This warrants serious efforts in developing effective cancer therapeutics. Many therapeutics have been attempted to target various types of cancers with each one having their own pearls and pitfalls. Surgery may sometimes be a solid choice for tumor removal but may not be feasible for liquid cancers. Also, significant variability in the outcomes after complete removal of the tumors followed with immunotherapy have been observed, such as in the case of brachial plexus tumors, glioblastoma, osteosarcoma, and others [11–16]. In situations where surgical resection is unable to be performed radiotherapy may be explored where a wide array of techniques may be used [17,18]. Effectiveness of HDR-brachytherapy was compared to stereotactic ablative therapies which are minimally invasive procedures that remove tumors using radiation or laser therapy. However, it was shown that the effectiveness of HDR-brachytherapy begins to dwindle for lesions that are greater than 3 cm as patient comfort starts to become an issue due to the high doses of radiation delivered [17,19]. Therefore, it must be known that this specific radiotherapy technique is hindered by the size of the lesion it can treat without sacrificing patient comfort. Another quite interesting rather intensive therapy that is also used is immunotherapy [18, 20–27]. The theory of this technique places heavy emphasis on the ability to train the immune system to fight cancer. Cancer immunotherapies may fulfil one of several roles including tumor escape and/or reactivation of antitumor immune responses, one example using targeted cytokines [15–18, 28–30]. Recently there has been a surge of therapies known as adoptive cell transfer therapies. This process is involved with obtaining the patient’s own immune cells to reprogram those cells to combat cancer. Cells are given the chimeric antigen receptor and aids T cells target and attach cancer cells. The multiplicative ability of these newly generated CAR-T cells makes this process unique with high levels of specificity to cancers upon infusion back into the patient. This may train the immune system to be active in preventing metastasis however, this in fact may not be as effective. In a process known as epithelial–mesenchymal transition (EMT) which plays a part in wound healing, tissue regeneration, and embryonic development, metastasis may occur leading to a resistance to both chemotherapy and immunotherapy [12,31–34]. Nonetheless, immunotherapy plays a key contributor in both immune-mediated cancer removal as well resistance to the immune response itself.

## CAR-T Cell and Approved Therapies

**Figure 1:**
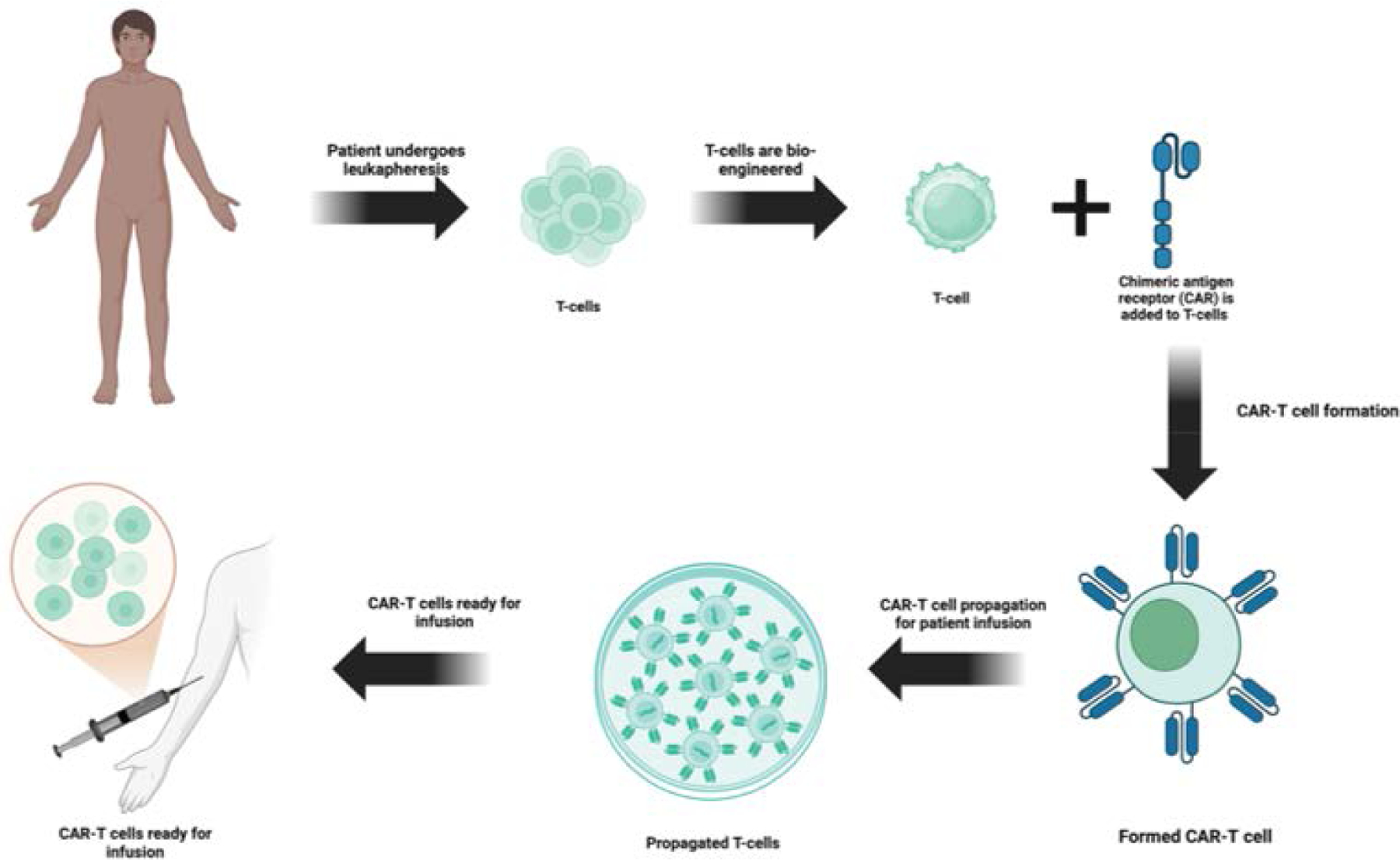
Process of CAR T-cell preparation for infusion in the patient.

**Figure 2:**
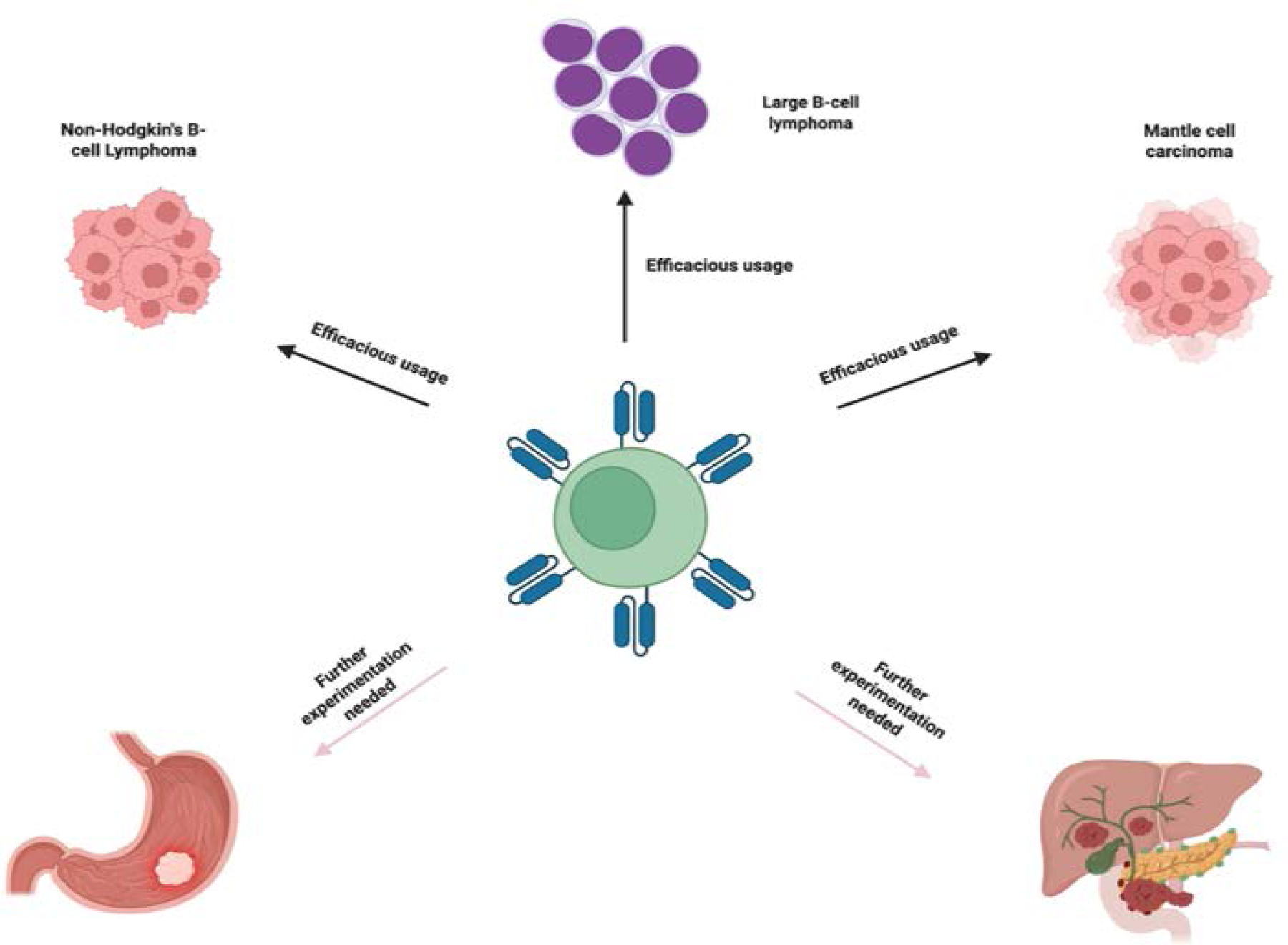
Examples of the cancers in which CAR T-cell therapy is efficacious. However, further studies are required in other cancers and indeed several clinical trials in solid tumors and autoimmune diseases are currently in progress.

CAR-T cell therapy has shown to have potential in treating liquid cancers and allow for custom designs to be created for higher specificity and efficacy as compared to other forms of cancer therapies and they usually contain an extracellular ligand binding domain, single chain variant fragment (scFv), a spacer domain, transmembrane domain and other cytoplasmic domains [35, 36]. ScFVs are major contributors to binding site recognition and future signaling, along with other aspects of the CAR structure may also play a dual role in both effectiveness as well as toxic effects. In the preparation of CAR T-cells, leukapheresis procedure is performed to collect patient’s blood T-cells, followed by genetic reengineering of the collected T-cells to produce chimeric antigen receptors (CARs) on the surface of T-cells so that CARs effectively recognize and bind to a specific antigen on the surface of cancer cells (Figure 1). The modified CAR T-cells are propagated to create billions of these specialized cells. Before infusing these cells back into the patient’s bloodstream, the immune system of the patient is prepared with a short course of chemotherapy to enhance the effectiveness of CAR T-cells (Figure 1). The infused CAR T-cells locate, attack, and destroy the cancer cells that express the target antigen. The infused CAR T-cells continue to multiply in the body of the patient and thus provide a long-term defense against the cancer. Chimeric antigen receptors (CARs) can be used as a quicker method to form active T-cells which can be used to target tumors as opposed to immunization methods [37]. Currently there are also several FDA approved CAR-T therapies some of which will be discussed in this article. Those include axicabtagene ciloleucel, brexucabtagene autoleucel, lisocabtagene maraleucel, tisagenlecleucel, idecabtagene vicleucel [38–42]. The FDA has approved the clinical use of CAR T-cell therapy in several cancers, including Non-Hodgkin’s B-cell lymphoma, diffuse and primary mediastinal large B-cell lymphoma, Mantle cell carcinoma, B-cell acute lymphoblastic leukemia, and multiple myeloma. However, Several clinical trials are currently in progress investigating the effect of CAR T-cell therapies in other types of cancer, including solid tumors like breast cancer and lung cancer, and autoimmune diseases (Figure 2).

## Mechanism of Action of CAR-T Cell Therapy

CAR-T is one of the main branches of adoptive T cell therapies where tumor cell killing occurs via activation of T-cells to thereby bind to the tumor cell surface antigens. The specificity of binding can be traced back to the scFv, spacer domain, and costimulatory domains. Upon binding, anti-tumor effects can be seen via the distribution of perforins, Fas ligands, and the release of cytokines to effectively attack the stroma surrounding the tumor [43]. This places CAR-T as beyond just a niche treatment regime but an ever-so-developing one that can be used alongside monoclonal therapies such as monoclonal checkpoint inhibitors [44]. The growth of CAR-T use has risen dramatically from the 2010s to now and especially in the last 2 years. With the estimated number of clinical trials with 1,580 as of April 2024 which rose up from 1,087 clinical trials reported in January 2023 [45, 46]. With the ever-growing usage, experimentation, and efficacy of CAR-T we must also explore the toxicities that accompany CAR-T and manage toxicities in patients. The toxicities I would like to explore in this paper are those of cardiovascular origin. Since many patients present with existing cardiovascular issues prior to any cancer therapeutics. Given that the age-standardization incidence rate for cardiovascular disease (CVD) has increased from 126.80 per 100,000 in 1990 to 129.85 per 100,000 in 2019, there becomes an increased need to limit CVD in general and cancer-presenting population as well as the overall global mortality rate of CVD is almost a third of the global population as mentioned by the W.H.O. (47). Thus, it is important to discuss such novel therapeutics despite the high levels of efficacy which will impact the global rise in CVD and cardiovascular health concerns world-wide. There is also a current need for toxicity management guidelines that need to be discussed and explored as well.

## Efficacy of CAR-T Cell Therapy in B-cell Lymphoma

Non-Hodgkin’s B-cell Lymphoma (NHL) is a type of lymphoma that manifests in several different variations ranging from highly aggressive to less aggressive NHL and indolent NHLs. Although there are several medications available in the forms of antineoplastics, steroids, and monoclonal antibodies, all of which have shown some effectiveness and durable results in patients, they do not provide cures or resistance to cancer relapse. Outcomes for patients struggling with NHL have been revamped with the introduction of CAR-T therapy for this type of cancer [14]. In a study using Lisocabtagene maraleucel (liso-cel) which is a CAR-T product, was used to determine effectiveness in relapsed or refractory large B-cell lymphomas. This study used the seamless design principle [48–50]. Patients were assigned three different dose ranges administered as two types: CD8+ and CD4+ CAR+ T cells. During the time of study from January 11, 2016 to July 5, 2019, 269 were treated with liso-cel. It was determined that safety and activity of liso-cel was independent of the dosage administered. Out of the 269 patients’ data from 256 patients were used to evaluate effectiveness and were a part of the efficacy-evaluable set. Of those 256, 186 had an objective response and 136 of those had a complete response. The most common adverse effect noted by this study was neutropenia in 161 patients out of 256 [51]. Although, a complete response was achieved a common problem with not just NHLs but other types of cancers as well is relapse. A follow up study was done two years later to assess the complete response rate. Liso-cel was shown to have 53% complete response rate where the median time to reach a complete response rate or a partial response was 0.95 months. A median duration of response was determined to be 23.1 months. Most notable adverse effects noted were neutropenia, anemia, and fatigue [52]. CAR-T modalities such as liso-cel have shown to lead to improved outcomes for patients diagnosed with aggressive and relapsing cancers. It has also improved the quality of care that is given to patients and become an integral part of cancer care in certain types of cancers such as NHL B-cell lymphoma. It now offers patients a novel type of therapeutic that is also in several ways personalized to the patient’s needs and has shown effectiveness as a last resort for patients.

## Efficacy of CAR-T Cell Therapy in Refractory Large B-cell Lymphoma

Another potential usage of CAR-T has been seen in therapeutic plans for refractory large B-cell lymphoma. In a multicenter phase 2 clinical trial, 101 patients diagnosed with various B-cell lymphomas including large B-cell lymphoma, primary mediastinal B-cell lymphoma, transformed follicular lymphoma all of which had refractory disease were dosed with the 2×106 anti-CD19 CAR T cells by the name of axi-cel. Objective response rate was noted to be 82% with a complete response rate of 54%. Most common adverse effects noted were neutropenia, anemia, and thrombocytopenia. In this study they claimed a dose-independent response with 3 noted deaths during treatment. Durability of the response rates were also mentioned, noting that after a follow up time of 1 year, the objective response rate was 82% with a complete response rate of 58% with a median duration of response was 11.1 months [52,53]. In a CD-22 directed CAR-T cell therapy a small Phase-I trial was conducted with 3 Large B-cell lymphoma patients who had relapsed CD-19 directed CAR-T therapy and were given at least 2 prior lines of therapy Large B-cell lymphomas showed complete remission [54]. Although, the sample size used was extremely small and not at all conclusive for the treatment itself. However, therapy was well-tolerated, and no toxicities were observed. This shows that CAR-T as a therapy can be used in conjunction with itself and be re-engineered and re-applied as a therapeutic intervention and CD-22 is another potential target that can be used for CAR-T therapy. However, it is still to be noted that the sample size was only 3 patients, and further research needs to be done to understand the full efficacy and toxicities associated with the treatments and how this therapy compares to CD-19 directed CAR-T therapy.

## Efficacy of CAR-T Cell Therapy in Mantle Cell Carcinoma

Mantle-cell carcinoma is a rare-subtype of B-cell NHL which arise from naive B-cells in the mantle zone of lymphoid follicles. Most patients with this type of cancer present with very advanced stages of cancers and lymphadenopathy and bone marrow dysfunction, generally considered to be very aggressive and incurable but have shown susceptibility to CAR-T therapy [55–57]. A study was conducted testing the effectiveness of a CAR-T modality on mantle cell carcinoma named Phase 2 TARMAC trial. Patients enrolled in this study were enrolled after 2 or more lines of treatments were employed including Bruton tyrosine kinase inhibitors (BTKi) which have shown poor results with additional additive therapies and as a stand-alone therapy. BTKi users had an overall median survival level of just 9.1 months in contrast to CAR-T which has an overall median survival of 32.1 months. BTKi has also shown limited capability of being able to improve response rates and durability with cancers when paired alongside other combination therapies [58]. However, the combination of BTKi and CD19 CAR-T cells have shown encouraging results in the TARMAC study. Twenty relapsed/refractory mantle-cell carcinoma patients were recruited for the study and were started on ibrutinib- a BTKi prior the leukapheresis process of CAR-T. Overall response rate was 80% and an overall survival rate of 100% for those measured 12 months after treatment and with only 25% of patients were shown to be relapse-free after 1 year. Results showed that CAR-T not only has the potential to improve response rates and median survival therapies on its own but has the potential to improve current standard of care therapies and enhance the efficacy of those to reach much improved rates of efficacy. However, one thing to note is that adverse events were also seen in all patients in the study that employed the treatment. Cytokine release syndrome (CRS) occurred in 75% of the patients with a median duration of 3–4 days- mostly of grade 1 and grade 2 toxicity. Two patients experienced low grade neurotoxicity. Grade 3–4 hematologic toxicities occurred in 75% of patients such as neutropenia being the main toxic effect experienced [59]. It is also important to note that there has not been a follow-up study to monitor the effects and toxicities in the patients in this study and further research is still required in the long term with regards to CRS in mantle-cell carcinoma patients treated with CAR-T.

## Efficacy of CAR-T Cell Therapy in Diffuse Large B-cell Lymphoma

In the diffuse large B-cell lymphoma (DLBCL) cases, a meta-analysis of 14 clinical trials was conducted with a total number of 419 patients with B-cell NHL lymphomas most of which were refractory or relapsed diffuse large cell B-cell lymphomas (306 patients with diffuse large cell B-cell lymphomas) [59]. The CAR-T constructs that were included were CD-19 targeted such as the following: axi-cel, tisa-cel, and liso-cel. Objective response rates were measured along with complete remission rates, 12-month progression free survival, overall survival and safety and toxicities of these therapies. An overall response rate was found to be 69% across the entirety of these studies with a 49% complete response rate. Among the 306 diffuse B-cell lymphoma patients the objective response rate was at 68%. Overall, CAR-T has shown variable but strong levels of durability with the median progression free survival being 4.5 months and an overall survival rate of 58% among all patients and a median overall survival rate of 13.2 months. CAR-T has shown strong results in relapsing NHL B cell lymphomas including diffuse large B- cell lymphomas. However, the safety profile was not well characterized, and results were shown to be quite vague indicating that there is a need to monitor the toxicities of CAR-T alongside the results of the treatment for accurate assessment of these therapies. Another key missing piece of data that is missing from many of these CAR-T studies as well as the ones mentioned previously is the lack of follow-up/long-term data analysis beyond 12 months [54, 61–63].

## Efficacy of CAR-T Cell Therapy in Solid Tumors Targeting Mesothelin

Although many of the clinical trials done with CAR-T therapies have been focused on liquid cancers, CAR-T has still been trialed in solid tumors, however not without challenges. There are several FDA-approved CAR-T modalities for blood cancer such axi-cel, tisa-cel, liso-cel, and breexu-cel with durable remission rates CAR-T therapies struggle to enter the tumor microenvironment and are more susceptible to relapse. The variability of antigens located on many solid tumors such as lung cancer tumors have presented challenges [64,65]. The cost of CAR-T treatment and toxicities remain challenging to the efficacy of CAR-T on solid tumors and next-gen chimeric antigen receptors have been discussed such as armored CAR constructs and dual-target CARs have been brought up [66,67]. Although early, CAR-T has shown some promising early results in solid tumors targeting mesothelin which is a molecule that is broadly expressed in many cancers such as various cancers such as lung, pancreas, breast, ovarian, and others [68]. CAR-T has been paired up with the anti PD-1 antibody treatment. PD-1 is a checkpoint inhibitor that is found in T-cells after encountering an antigen, and it has immunosuppressive capabilities when it is engaged with PD-L1 the programmed cell death ligand 1 which can be found in the tumor microenvironment. The combination of PD-1 and PD-L1 has partnered well with monoclonal antibodies in a variety of cancers to improve antitumor activity [69,70]. Other techniques have implemented the use of CRISPR- associated protein 9 (Cas9) system alongside the usage of PD-1 axis blocking on CAR-T cells to achieve improved antitumor action in some preclinical work [71–74]. This shows that CAR-T therapy can also be combined with the Cas9 system to improve efficacy as well as be a strong therapy to use in conjunction with others. This shows the level of flexibility CAR-T can be used with in conjunction to other therapies. In another study the clinical effects were in fact tested. The Cas9 system was used to knock out endogenous TCR receptors to prevent mispairing and improve safety while a genetically engineered TCR was implemented in the cells. As a result, there was no toxicity seen in the patients with refractory cancers and there was one patient that exhibited some tumor regression showing the level of safety of this method as well as the potential for therapeutic effects out of a total of 3 patients [75]. The co-use of CAR-T and Cas9 were used in the first in-human Phase 1 clinical trials using mesothelin-targeted CAR-T cells. A total of 15 patients received dose-escalated CAR-T cells without the usage of lymphodepleting chemotherapy due to risk of uncontrolled CAR-T expansion and for risk of infection and lesion biopsies were collected 2–4 weeks after the infusion events. Safety assessments were made, and no unexpected adverse events occurred due to the dosage, and no CRS and toxicities were observed. There was one instance of a patient with Grade 3 pericardial effusion due to CAR-T infiltration however that was resolved with intervention and in general this technique of Cas9 engineered CAR-T cells showed to be well-tolerated in the patient population [74]. In terms of the efficacy 2 of the 15 patients reached stable disease states and no tumor shrinkage and a major decline in CAR-T levels were noted in this study and showed overall poor persistence and minimal efficacy. However, further studies may show how this may differ with and without lymphodepletion and how CAR-T therapy may respond in each of those conditions [74].

## CAR-T Targeting Pancreatic Cancer

Pancreatic ductal adenocarcinoma (PDAC) is known to be one of the most aggressive forms of cancer that presents with high mortality rates and poor prognoses. It also presents as a cancer that has high and aggressive rates and forms metastasis with median survival times being less than 1 year and with 10% or less of people with PDAC surviving 5 or more years [76]. In a study, patients with PDAC were given CAR-T therapy targeting claudin 18.2, a marker present in many PDAC patients and a tight junction marker [77,78]. The 3 heavily pre-treated patients with advanced claudin 18.2 PDAC patients were treated with CT041, a CAR-T therapy targeting claudin18.2 specifically. The patients enrolled were previously treated with at least one type of therapy prior to CAR-T infusion. The results of this study showed 1 complete remission and 2 partial remissions with the duration being at least 6 months of remission. CAR-T presence was also detected for 120 days supporting the durability of this study and grade 1–2 CRS only were observed. Despite a 100% objective response rate the sample size is very small, made up of just 3 people and long-term safety was not discussed [79]. The data of another clinical trial was also pooled in this analysis with the two studies being CT041-CG4006 and CT041-ST-01 [79]. The pooled analysis included 24 patients with metastatic, refractory pancreatic cancer with every patient receiving at least one prior line of therapy. These studies showed an overall response rate of 16.7% and medial overall survival time of 10 months with a 9.5-month median duration of response time. The toxicities observed were mostly CRS grade 1–2 along with Grade 3 or more hematological toxicities due to lymphodeletion. Although CRS grading did not reach past stage 2, the same size was very small (24 patients) lymphodepletion was a major contributor to hematological toxicity and durability of these studies still need to be established along with safety and efficacy [79,80]. CAR-T has also shown potential in tumor regression ability. In a single person case report a patient with advanced metastatic pancreatic cancer which was unresponsive to several lines of therapies was infused with CAR-T cells targeting claudin 18.2 following lymphodepletion [81]. Results showed complete remissions with rapid levels of tumor regression and the treatment remained durable with no remission after a follow-up of 8 months. However, in-situ PDAC relapse was seen at 8 months with a claudin 18.2 negative form of cancer. Toxicities also included fever, abdominal pain and nausea which were symptomatically treated [82]. Albeit a single person sample size, complete remission was reached however, questions remain regarding long-term toxicity and follow-up time. However, the early success for CAR-T in solid tumors in advanced metastatic pancreatic cancer does also demonstrate the efficacy of CAR-T as a therapeutic modality.

## CAR-T Treatment of Claudin-18.2-positive Gastro-esophageal Cancers

In a very recent study gastric cancers were evaluated that were positive for Claudin-18.2 isoform. 100 patients were treated with Satri-cel CAR-T therapy vs physician’s choice chemotherapy. Progression-free survival (PFS) and overall response rate (ORR) were measured. Satri-cel showed statistically significant improvement in PFS compared to chemotherapy. Toxicities were also mentioned in the abstract mentioning that toxicity remained controlled, but details were not mentioned. Overall, the study claimed Satri-cel to be improved over standard care in gastric cancer [81]. In a single person case report, a heavily pretreated metastatic gastric cancer patient was given two infusions of CT041 which is a claudin 18.2-targeted CAR-T therapeutic with lymphodepletion. Results showed a partial response by week 4 after first infusion and a complete response of the target after the second infusion for 8 months with minimal ascites. Following this therapy the patient regained energy life, and they were able to go back to taking care of their kids with an overall vastly improved quality of life after treatment. Toxicities were limited to Grade 2 CRS and grade 3 lymphopenia which likely resulted from the lymphodeletion [83]. Again, the sample size remains a concern as well as the durability as no data was provided after the follow-up time of 8 months and there remains a need for managing toxicities and garnering more data in the form of larger and more controlled studies.

## CAR-T Cell Therapy-associated Cardiovascular Toxicities

Despite the many advances made by CAR-T in treating hematological cancers, particularly in B-cell lymphomas and relapsing B-cell lymphomas [54,63]. However, it also may present as cardiotoxicity in patients. Several cardiac malignancies may include arrhythmias, heart failure, myocarditis, and many others while many manifest as cytokine release storms [84,85]. Cytokine release syndrome (CRS) is defined as the delayed onset of symptoms and usually occurs sometime after treatment. This differs from cytokine storms (CS) since CS occurs in a rapid fashion usually acting within minutes to hours [86]. The grading of CRS has been outlined into grade 1–4 with the severity of hypotension and hypoxia increasing from grade 2 onwards requiring increasing degrees of vasopressors and types of nasal airflows for hypoxic severities [87]. As CAR-T therapies begin to show efficacy and more widespan usage especially as the newly developing standard of care, the accurate classifying of CRS is needed [87,88].

## Biomarkers and Monitoring of Cardiotoxicity

This ushers in the need for thorough long-term monitoring in patients requiring CAR-T therapies and perhaps a need for co-treatment with therapies that explore cardioprotective measures associated with administering CAR-T therapy [89]. Two of the key cytokines which mediate CRS toxicity are IL-1 and IL-6 that may be released upon CAR-T infusion [90]. In particular, IL-6 has shown to exhibit long-lasting increase in vascular permeability and damage of the endothelial barrier which may lead to myocardial edema, coronary microvascular dysfunction, and vascular leak for example [91,92]. IL-6 has also been directly shown to have correlations to heart failure and reduced LVEF which presents as a higher risk of mortality overall [93,94]. In a meta-analysis study, the breakdown of cardiovascular associated CAR-T toxicity measured the data from 23 studies, with an unspecified number of patients to note. The results showed the most common cardiovascular events that occurred were arrhythmias which occurred in over half the patients and heart failure and cardiomyopathy combined making up an additional 50%, and to a much lesser degree acute coronary syndrome and cardiac arrest. It is also important to mention that patients that had these events were also subject to higher incidence of CRS grade 2 and higher [95]. It is also important to note that many patients may have experienced multiple symptoms however a breakdown of more detailed co-morbidities may be important for further context were not provided. While there are no currently official treatment modalities in place now there has been some efforts to reduce CRS and decrease cardiotoxicity. There has been some use of Tocilizumab in response to Grade 2 or higher CRS to block IL-6 in CD-19 CAR-T therapy and has shown efficacy in limiting duration of CRS in patients as well as used as prevention for CRS in liquid cancers [96,97]. The exploration of a treatment modality for preventing CAR-T related cardiotoxicity with the implementation of echocardiographic and biomarker evaluations at pre-specified time points likely lead to more improved monitoring of cancer therapy-related cardiac dysfunction (CTRCD) showing that both imaging as well as biomarker testing could decrease the likelihood of CRS and its relationship to cardiotoxicity due to CAR-T therapy, with the key step being early monitoring [98].

## Treatment of Cytokine Release Storm

Treatment modalities that have been used to treat CRS have been grade specific. Any patient who presents to the hospital with CRS first undergoes monitoring of vital signs, neurological assessments and blood counts, and biomarkers [99]. Grade 1 CRS management is usually with the management of symptoms such as fever reduction with acetaminophen as well as appropriate antibiotic usage and fluid balance maintenance. Tocilizumab can be added starting at grade 2 onwards while adding vasopressors for treatments with Grade 3 and higher [100]. With Grade 2 and higher CRS is a strong predictor of cardiotoxicity [101–103].

## Risk Factors for CAR-T-induced Cardiotoxicity

CAR-T associated cardiomyopathy may be due to several causes noting vascular leak and it is not uncommon for patients to suffer from capillary leak syndrome following CAR-T therapy [104]. Capillary leak syndrome (CLS) has also been correlated with hypotension and is like CRS in that IL-6 is a common cytokine that is released in CLS which pertains to cardiomyopathy [105]. Cardiotoxicity and cardiovascular toxic events due to the CAR-T have been studied in CD-19 CAR-T cell therapy mainly which has been used for hematological cancers have commonly presented patients with arrhythmia and heart failure [106,107]. It is important to note that this data comes from small sample sizes and from the more well-studied types of CAR-T therapies. Cardiotoxicity amongst CAR-T therapy receiving patients was measured and showed a statistically significant difference in the incidence of Cardiovascular events with elderly patients having higher incidences than younger adults and differences between genders were not studied in lymphoma cancers [108]. CAR-T associated cardiotoxicity has also been observed in the pediatric and young adult population reporting hypotension and the use of vasopressors in 25% of the study population [109]. However, it was noted that at the 6-month follow up period patients that experienced left ventricular systolic dysfunction had recovered however, risk factors for cardiotoxicities included: prior history of systolic or diastolic dysfunction and previous cardiomyopathy and levels of radiation did not correlate with cardiotoxicity in these patients [110–117]. In a patient population treated with CD-19 CAR-T therapy, elevated troponin levels were noticed with older patients that presented with common cardiovascular risk factors such as diabetes, hypertension, hyperlipidemia, etc. Higher troponin levels were also observed in accordance with higher CRS grades and cardiotoxicity [107]. However, further research has shown that B-type natriuretic peptide (BNP) might be a better and more sensitive early cardiac biomarker for cardiotoxicity in early stages for hemodynamic distress [118]. Patients given lymphoma targeted CAR-T cell therapy, the results showed a statistically significant difference between elderly patients compared to adults that experienced adverse cardiovascular impacts following CAR-T therapy. The most featured adverse cardiovascular events featured arrhythmia and heart failure [108]. In a CAR-T treated relapsed or refractory aggressive B-cell lymphoma similar results were found showing that the older populations were more susceptible to adverse cardiovascular events due to CAR-T therapy particularly those with pre-existing cardiovascular disease [110, 116]. Overall, research is showing that elderly patients, particularly those that demonstrate prior cardiovascular dysfunction are more prone to cardiovascular toxicity. Additionally, CAR-T induced cardiotoxicity is related primarily to the presence and severity of CRS. Lastly, the exact mechanism of CAR-T therapy cardiotoxicity has not yet been identified; however, it has been proposed that some occurrence of off-site cross-reactivity between T cells and titin may be part of the issue [110,111]. Studies have also shown that pediatric and young adult patients seem more likely to have reversible cardiotoxicity than adults especially with those that have CRS grades of 2 or higher [85].

## Future Directions for CAR-T-induced Cardiotoxicity

The recent advancements in CAR-T as well as the emphasis on treating relapsing cancers has made a great leap into the realm of personalized medicine which has opened the door for many ground-breaking clinical therapies but not without the presence of cardiotoxicity. Currently there is no standardized way of evaluating the cardiac function of a patient prior to treatment and many of these methods are dependent on the institutions conducting these studies [113,114]. To properly manage these cardiovascular events from CAR-T the American Society of Clinical Oncology (ASCO) have developed some clinical practice guidelines that pertain to cardiotoxicity in anti-cancer therapies [115]. Additionally, the University College of London Hospital has also come up with several guidelines on how to manage and plan for treatment for CAR-T cell therapy to conduct risk assessment for patients who will be treated with CAR-T to screen for an in-depth cardiovascular history as well as hypertension, diabetes, obesity, and smoking [116,117]. Additionally, biomarkers such as BNP and troponin may be measured and rises in both biomarker levels may be anticipated for early monitoring. Imaging stress tests may also be considered prior to CAR-T infusion as well [115]. Although very early, CardioMEMs may also be considered, and further research is to be conducted regarding the use of this device in the context of CAR-T induced cardiotoxicity which is a device used to manage heart failure prior to symptoms occurring. With the rise in use of CAR-T therapy, and with regards to the efficacy of the therapeutic modality in treating hematological cancers and with early development in solid cancers as well, early monitoring is needed to prevent cardiotoxicity. There are still several aspects such as cardiotoxicity that still need to be improved for CAR-T to be able to reach its full potential as a strong cancer therapy of choice. While the cardiotoxicity of CAR-T may remain acute, many patients receiving CAR-T therapy may already have pre-disposed cardiovascular conditions along with prior forms of treatment done as well raising the risk of cardiovascular toxicity. This ushers in the need for thorough long-term monitoring in patients requiring CAR-T therapies and perhaps a need for co-treatment with therapies that explore cardioprotective measures associated with administering CAR-T therapy. There is also a need for further research to be conducted in managing the CRS symptoms to further limit CRS and its associated cardiotoxicity. While the bulk of the CAR-T associated cardiotoxicity research has focused on the short-term cardiotoxic effects of CAR-T therapy, the long-term cardiovascular effects of CAR-T have been sparsely researched. Especially, as the growth of CAR-T usage speeds up, the results of future clinical data regarding long-term effects of this highly efficacious cancer treatment modality may dictate how it is managed and screened prior to its implementation in patients to receive the best possible results for patients.

## Key Points

- CAR T-cell therapy is indeed a significant advancement in cancer treatment with impressive efficacy in treating certain hematological malignancies with long-term remission and even cure,
- The infused the CAR T cells locate and attack cancer cells that express the target antigen, leading to their destruction.
- The CAR T cells may multiply in the body of the patient, providing a long-term defense against the cancer.
- CAR T-cell therapy has been approved by the FDA in the treatment of several cancers, including Non-Hodgkin’s B-cell lymphoma, diffuse and primary mediastinal large B-cell lymphoma, Mantle cell carcinoma, B-cell acute lymphoblastic leukemia, and multiple myeloma.
- Clinical trials are currently in progress investigating the effect of CAR T-cell therapies in other types of cancer, including solid tumors like breast cancer and lung cancer, and autoimmune diseases.
- CAR T-cell infusion can induce severe adverse effects, including cytokine release storm, cardiotoxicity, immune effector cell-associated neurotoxicity syndrome, anemia, thrombocytopenia, neutropenia, and infections due to weakened immune system.
- Some patients with CAR-T cell infusion experience mild to life-threatening cardiovascular complications, such as arrhythmias or heart failure, due to the inflammatory response.
- The activated T-cells following CAR T-cell infusion may release inflammatory cytokines and trigger systemic inflammatory response, causing symptoms like fever, low blood pressure, and organ dysfunction.
- Heterogeneous clinical presentation of CAR-T adverse events underscores the need for vigilant monitoring for cardiotoxicity and individualized management strategies.
- Careful clinical studies are warranted for long-term efficacy and safety.
